# Low Caloric Intake Confers Cardiovascular Protection and Improves Functional Capacity Without Affecting Immunological Response in Sedentary Older Adults

**DOI:** 10.3390/nu16213677

**Published:** 2024-10-29

**Authors:** Meiry de Souza Moura-Maia, Boris Brill, Rosa Helena Ramos Paula-Vieira, Nycole Vieira Ramos-Gomes, Dobroslav Melamed, Anamei Silva-Reis, Eduarda Teodora Rachid Wolpp, Naiara Nadia Moreira-Silva, Yanesko Fernandes Bella, Rodolfo P. Vieira

**Affiliations:** 1 Laboratory of Pulmonary and Exercise Immunology, Universidade Evangélica de Goiás (UniEvangélica), Avenida Universitária Km 3,5, Anápolis 75083-515, GO, Brazil; meiry@unievangelica.edu.br (M.d.S.M.-M.); rosahelenarpv@yahoo.com.br (R.H.R.P.-V.); nycole.gomes@unievangelica.edu.br (N.V.R.-G.); anameisreis97@gmail.com (A.S.-R.); eduwolpp@gmail.com (E.T.R.W.); naiara.silva@unievangelica.edu.br (N.N.M.-S.); 2 Leniado Medical Center, Divrei Khayim St. 16, Nethanya 4244916, Israel; drborisbrill@gmail.com; 3 Libi Pharm, Department of Research and Development, Ben Gurion 70, Rechovot 7639461, Israel; dobroslav.melamed@gmail.com; 4 Heroes Science Institute, Rua Ezequiel Freire, 51, Room 107, São Paulo 02034-000, SP, Brazil; yanesko@hotmail.com; 5 Post-Graduate Program in Human Movement and Rehabilitation, Federal University of Sao Paulo, Rua Talim 330, São José dos Campos 12231-280, SP, Brazil

**Keywords:** hypocaloric diet, caloric deficiency, cardiovascular hemodynamic, aging, cardiac function

## Abstract

Background: Aging is characterized by a decline in the cardiovascular hemodynamic response, which may be aggravated by undernutrition. However, no study has evaluated whether low caloric intake may affect cardiovascular hemodynamics and its possible relation with functional capacity and immune response in older adults. Methods: Sixty-one older adults of both genders were enrolled in this study and were classified as normocaloric (n = 18) and hypocaloric (n = 43). All volunteers were evaluated for cardiovascular hemodynamics using impedance cardiography (PhysioFlow^®^); functional capacity by the 1′ sit-to-stand test with SpO_2_ monitoring; whole-blood analysis using an automated hematocytometer (Sysmex^®^); and levels of IL-6, TNF-alpha, IL-10, and Klotho by ELISA. Results: The hypocaloric group presented impaired functional capacity, measured by a reduced number of sit-to-stand repetitions (*p* < 0.0251) and impaired delta of SpO_2_ (*p* < 0.0307). In contrast, the hypocaloric group presented an improved stroke volume (*p* < 0.0352), systemic vascular resistance (*p* < 0.0075), and systemic vascular resistance index (*p* < 0.0184). In addition, no changes were observed in the whole-blood analysis (*p* > 0.05) or for IL-6 (*p* > 0.05), TNF-alpha (*p* < 0.05), IL-10 (*p* < 0.05), and Klotho (*p* > 0.05). Conclusions: A long-term hypocaloric diet in eutrophic older adults’ resulted in an enhanced cardiovascular hemodynamic response but was associated with reduced functional capacity without changes in the immune response.

## 1. Introduction

The impact of caloric protein undernutrition on cardiovascular hemodynamics has been reported since 1977, particularly in children [1]. At this time, a hallmark study demonstrated that severely undernourished African children presented with bradycardia and hypotension and an impaired cardiac index, stroke index, and heart work, beyond intravascular volumes [1]. Research findings suggest that there is a high prevalence of undernutrition occurring alongside chronic heart failure (HF), resulting in various adverse outcomes, especially in older adults [2]. The recommendations outlined by the European Society of Cardiology (ESC) regarding the management of acute and chronic HF emphasize the importance of monitoring and mitigating undernutrition in patients with HF [2]. Studies demonstrate a frequent co-occurrence of undernutrition and chronic HF. The prevalence of undernutrition in individuals with this condition is as high as 69%, irrespective of age, gender, or left ventricular ejection fraction [3]. This phenomenon is likely attributed to underlying hemodynamic shifts and an inflammatory reaction [4]. Research indicates that 80% of malnourished patients experience mortality or require rehospitalization within 12 months, which is in contrast to 30% of patients maintaining a normal nutritional status [4].

The natural and not pathological aging process already induces cardiovascular changes that predispose an individual to the development of heart failure [5]. Heart failure can be conceptualized as the ultimate culmination of the aging process within the cardiovascular system, symbolizing the merging of age-related alterations in the cardiovascular structure and function and aging-related changes in other bodily systems, compounded by the escalating incidence of cardiovascular ailments among the elderly population [5]. The aging heart experiences several functional alterations and compensatory mechanisms that impair its capacity to adapt to heightened work demands and reduce its reserve capacity [5]. These include modifications in the maximal heart rate, end-systolic volume (ESV), end diastolic volume (EDV), contractility, prolonged systolic contraction, prolonged diastolic relaxation, sympathetic signaling, and more [5]. Beyond that, the inflammatory response plays a key role in such cardiovascular alterations, which are characterized by increased levels of pro-inflammatory cytokines, such as IL-6 and TNF-alpha, as well as reduced levels of anti-inflammatory mediators, such as IL-10 and Klotho [6].

Therefore, it is unclear whether older adults experiencing caloric undernutrition but exhibiting a “healthy status”—defined by the absence of chronic heart diseases and full independence in daily activities—show any alterations in cardiovascular hemodynamic responses. This study tested the hypothesis that nutritional status may influence cardiovascular hemodynamic responses in older adults.

## 2. Material and Methods

### 2.1. Patient Recruitment and Study Design

Sixty-one older adults of both genders were recruited from the community of Anápolis city, state of Goiás, Brazil. From these initial 78 older adults, 61 were enrolled into this study, since they satisfied the inclusion (free and voluntary participation in the study; signing of the consent form; availability to participate in the 2 evaluations; and sedentary for at least 1 year) and exclusion (no presentation of any neurological impairments to avoid the evaluations not being performed properly) criteria. After the nutritional evaluation, the older adults were classified into normocaloric, hypocaloric, and hypercaloric. This study was approved by the Ethical Committee of the Evangelical University of Goiás (UniEvangélica) and registered under number 6.095.284 on 1 June 2023. In addition, the study protocol was pre-registered at Plataforma Brazil (https://plataformabrasil.saude.gov.br/login.jsf, accessed on 30 June 2023) with number 68189923.5.0000.5076 on 24 May 2023.

### 2.2. Nutritional Evaluation

The nutritional evaluation was performed using the 24 h food record [7], in addition to a food frequency questionnaire [8]. This evaluation allowed us to classify the older adults into hypocaloric, normocaloric, and hypercaloric. Both questionnaires recorded details such as which type of food was consumed, as well as the amount of each food that was consumed, in the last 24 h and the frequency of this consumption. With this information, we were able to calculate the exact quantity of calories, proteins, carbohydrates, and fat consumed every day by the volunteers, allowing us to classify them as hypocaloric, normocaloric, and hypercaloric.

### 2.3. Physical and Clinical Characterization

All volunteers were weighed (kilogram; kg) and measured (meters; m) using a mechanical scale with a stadiometer (Welmy^®^, Sao Paulo, Brazil), and their body mass index (BMI; kg/m^2^) was calculated [9]. Their blood pressure (millimeter of mercury; mmHg) and heart rate (beats per minute; bpm) were evaluated using an arm blood pressure digital device (HEM-7122^®^, Omron^®^, Kyoto, Japan), with these measurements being carried out at rest in a sitting position [9]. The body composition was assessed by using a tetrapolar bioimpedance (HBF 514C^®^, Omron^®^, Japan), which measured the fat mass and lean mass, presented in percentage (%) and in kilogram (kg). The waist and hip circumference were measured using a professional tape measure in centimeters and millimeters. The waist-to-hip ratio was calculated by dividing the waist by the hip circumference. Similarly, the right and left calf circumferences were measured as well. The hand grip strength was measured using a hydraulic hand dynamometer (Jamar, Lafayette Instrument Company, Lafayette, LO, USA). The results were expressed in kilogram (kg).

### 2.4. One-Minute Sit-to-Stand Test with O_2_ Saturation

The 1 min sit-to-stand test is designed to evaluate lower limb strength, endurance, and functional fitness in the elderly population [10]. This test is commonly used to assess an individual’s ability to perform repeated sit-to-stand movements, which are crucial for daily activities [10]. The volunteers were instructed to stand up fully and sit down as many times as possible within one minute, without using their arms for support. Additionally, the partial oxygen saturation (SpO_2_ %) was monitored before, during, and after the test. The results were expressed in the number of sit-to-stand movements performed in 1 min, and the initial and final SpO_2_ (%) was recorded. The delta (initial SpO_2_ minus final SpO_2_) was calculated.

### 2.5. Cardiovascular Hemodynamic Parameters

Cardiovascular hemodynamic parameters at rest were obtained using the Physioflow^®^ equipment (Bristol, TN, USA), which is an impedance cardiography system. This system provides continuous, accurate, reproducible, and sensitive measurements of the following parameters: heart rate (HR; bpm); stroke volume (SV; mL); stroke volume index (SVI; mL/m^2^), which corresponds to the stroke volume corrected for body mass; cardiac output (CO; L/m); cardiac index (CI; L/m/m^2^); systemic vascular resistance (SVR); systemic vascular resistance index (SVRI; d·s/cm^5^·m^2^); left ventricular stroke work index (LVSWI; kg·m/m^2^); ejection fraction (EF; estimated %); end diastolic volume (EDV; mL); thoracic fluid content (TFC); and end diastolic function ratio (EDFR) [11]. With the volunteer in a supine position, six electrodes are placed according to the manufacturer’s specifications for device calibration. After electrode placement, a 1 min stabilization period is implemented for signal stabilization. Once the signal is stable, signal recording begins and continues for a period of 3 min [11].

### 2.6. Blood Collection and Cytokine Analysis

The analysis focused on evaluating systemic inflammation and the immune response. Venous blood samples (5 mL) were collected in sterile vacuum tubes containing EDTA K3 as an anticoagulant. Whole-blood analysis, including platelet count and white and red blood cell counts, was immediately performed on 25 µL of the sample using a Sysmex 800i analyzer (Sysmex, Kobe, Japan). The remaining blood was centrifuged at 1000× *g* for 7 min at 4 °C, and the plasma was subsequently stored at −86 °C. Interleukin 6 (IL-6) (DY206), IL-10 (DY217B), TNF-α (DY210), and Klotho (DY5334-05) levels were measured using DuoSet ELISA kits (R&D Systems, Minneapolis, MN, USA), and the readings were performed using a Spectramax I3 microplate reader (Molecular Devices, San Jose, CA, USA) [12].

### 2.7. Statistical Analysis

GraphPad Prism 5.0 software (GraphPad Software, CA, USA) was used for statistical analysis and graph construction. Data normality was assessed using the Kolmogorov–Smirnov test. Since all data presented parametric distributions, the Unpaired Student’s *t* test was used to compare the normocaloric and hypocaloric groups. A *p* value of less than 0.05 was considered statistically significant.

## 3. Results

### 3.1. Nutritional Evaluation

From the 78 older adults who volunteered for the study, 66 fulfilled the inclusion criteria, and after the nutritional evaluation, they were classified as normocaloric (n = 19), hypocaloric (n = 44), and hypercaloric (n = 3) according to their 24 h food records [7] and the food frequency questionnaire [8].

### 3.2. Physical and Clinical Characterization

As shown in Table 1, there are no differences among the groups regarding age (*p* = 0.7239), height (*p* = 0.9348), systolic blood pressure (*p* = 0.9414), diastolic blood pressure (*p* = 0.8055), heart rate (*p* = 0.1648), waist-to-hip-ratio (*p* = 0.5057), right calf circumference (*p* = 0.5282), or left calf circumference (*p* = 0.9526), which shows a homogeneity between the normocaloric and hypocaloric groups. However, the body weight (*p* = 0.0039), body mass index (*p* = 0.0027), fat mass (*p* = 0.0086), lean mass (*p* = 0.0448), waist circumference (*p* = 0.0027), and hip circumference (*p* = 0.0009) were different among the normocaloric and hypocaloric groups.

### 3.3. The 1′-Sit-to-Stand Test with O_2_ Saturation

**Figure 1:**
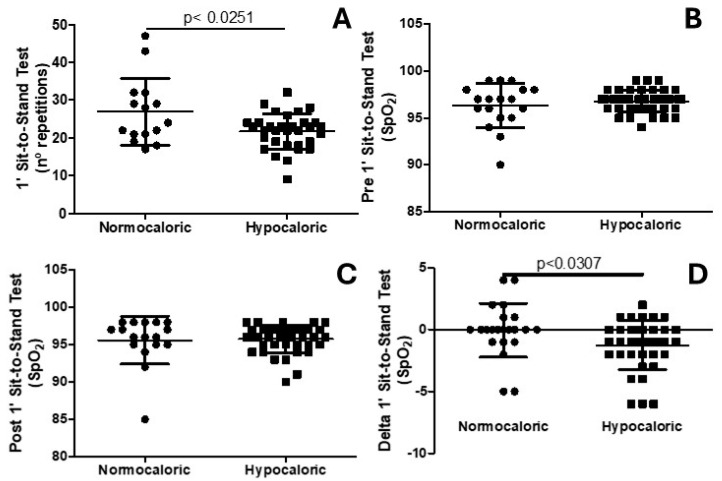
(**A**) shows the number of repetitions in the 1′ sit-to-stand test. (**B**) shows the partial oxygen saturation (SpO_2_) at rest. (**C**) shows the partial oxygen saturation (SpO_2_) after the 1′ sit-to-stand test. (**D**) shows the variation (delta) inf partial oxygen saturation (SpO_2_) considering the results before and after the 1′ sit-to-stand test.

Figure 1 shows that older adults under a hypocaloric diet presented worse performances on the 1′-sit-to-stand test, presenting a smaller number of sit-to-stand repetitions (*p* < 0.0251; Figure 1A), which was followed by an impaired delta of SpO_2_ (*p* < 0.0307; Figure 1D). Importantly, no differences in SpO_2_ at resting (*p* = 0.3064) and immediately (*p* = 0.8098) after the sit-to-stand test were observed when comparing the normocaloric group with the hypocaloric group.

### 3.4. Cardiovascular Hemodynamics

**Figure 2:**
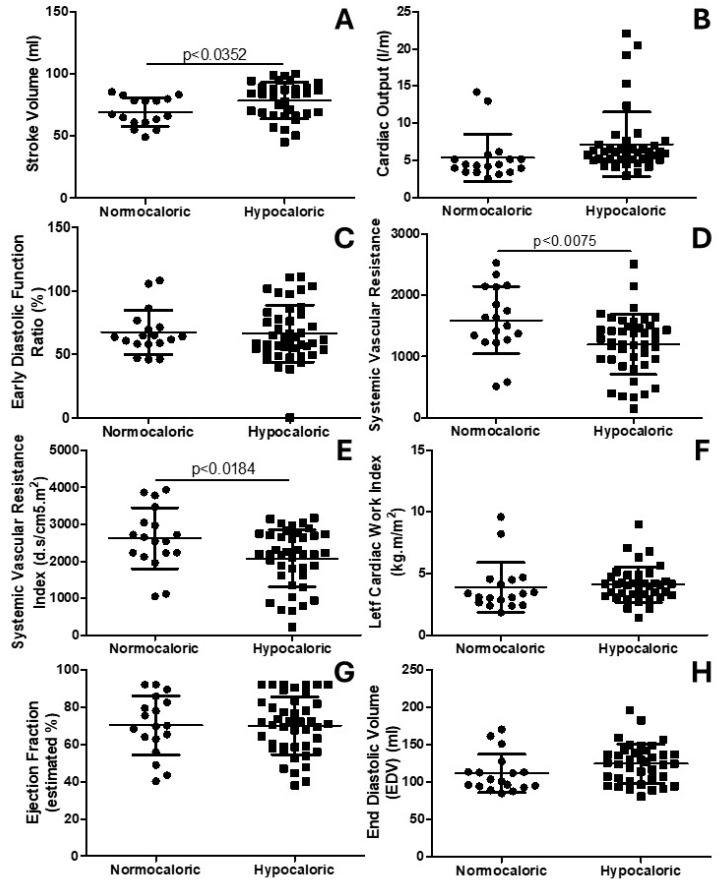
The parameters of cardiovascular hemodynamics. (**A**) shows the stroke volume. (**B**) shows the cardiac output. (**C**) shows the early diastolic function ratio. (**D**) shows the systemic vascular resistance. (**E**) shows the systemic vascular resistance index. (**F**) shows the left cardiac work index. (**G**) shows the ejection fraction. (**H**) shows the end diastolic volume.

Figure 2 shows the cardiovascular hemodynamic parameters. Figure 2A shows an increased (*p* = 0.0352) stroke volume for the hypocaloric group compared to the normocaloric group. Figure 2B shows no differences (*p* = 0.1129) in cardiac output between the normocaloric and hypocaloric groups. Figure 2C shows no differences (*p* = 0.8719) in early diastolic function ratio between the normocaloric and hypocaloric groups. Figure 2D shows that the hypocaloric group presented a reduced systemic vascular resistance (*p* = 0.0075) compared to the normocaloric group. Similarly, Figure 2E shows that the hypocaloric group presented a reduced systemic vascular resistance index (*p* = 0.0184) compared to the normocaloric group. Figure 2F shows no difference (*p* = 0.6006) in the left cardiac work index when comparing the normacaloric with the hypocaloric group. Indeed, Figure 2G shows no difference (*p* = 0.9850) in the ejection fraction between the normocaloric and hypocaloric groups. Figure 2H shows no difference (*p* = 0.0805) in the end diastolic volume between the normocaloric and hypocaloric groups.

### 3.5. Whole-Blood Analysis

**Figure 3:**
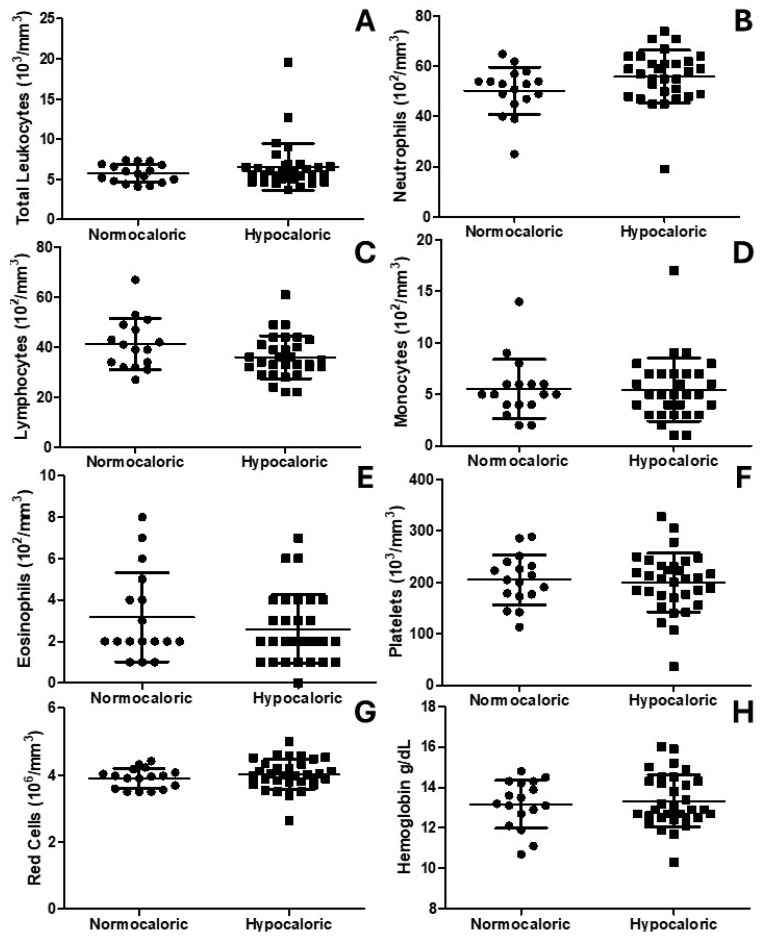
Diagrams showing the whole-blood analysis. (**A**) shows the total leukocytes. (**B**) shows the neutrophils. (**C**) shows the lymphocytes. (**D**) shows the monocytes. (**E**) shows the monocytes. (**F**) shows the eosinophils. (**F**) shows the platelets. (**G**) shows the red cells. (**H**) shows the hemoglobin.

Figure 3 shows the leukogram, platelets, red blood cells, and hemoglobin. Figure 3A–E show that the hypocaloric group does not exhibit any differences in total and specific leukocytes. In addition, Figure 3F shows no differences in the number of platelets between the normocaloric and hypocaloric groups. Lastly, Figure 3G,H show no differences in red blood cells or hemoglobin, respectively.

### 3.6. Systemic Levels of Cytokines

**Figure 4:**
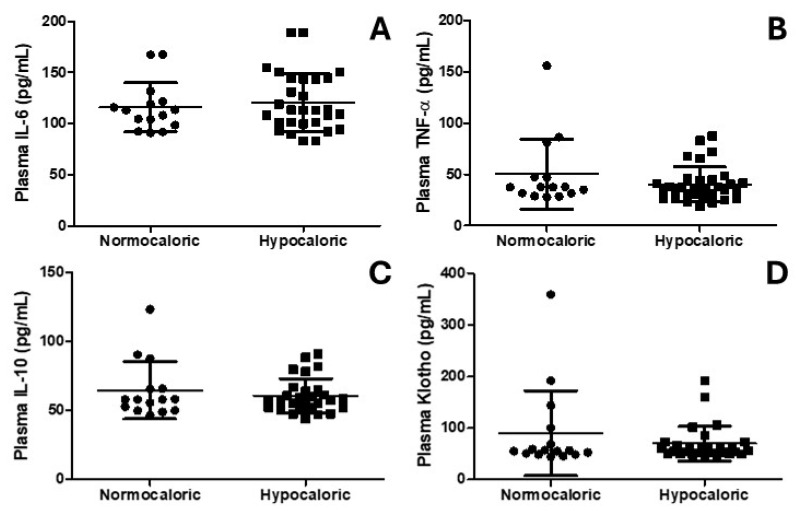
Diagrams showing the plasma cytokine analysis. (**A**) shows the plasma levels of IL-6. (**B**) shows the plasma levels of TNF-alpha (TNF-α). (**C**) shows the plasma levels of IL-10. (**D**) shows the plasma levels of klotho.

Figure 4 shows the plasma levels of IL-6, TNF-alpha, IL-10, and Klotho. Interestingly, Figure 4A–D show no differences in the levels of IL-6, TNF-alpha, IL-10, and Klotho, respectively.

## 4. Discussion

The present study investigated, for the first time, whether undernutrition in older adults could influence their cardiovascular hemodynamics and functional response and whether such an influence would be a response to possible alterations in the immune response. In this context, this study revealed that undernutrition in older adults positively influenced their cardiovascular hemodynamics, but on the other hand, it impairs their functional capacity, demonstrated by a reduced number of 1′-sit-to-stand tests, as well as impaired oxygen desaturation. In addition, this study demonstrated that such responses did not occur in response to any changes in the cellular and humoral immune responses.

The 1′-sit-to-stand test is a reliable test and widely used to evaluate the functional capacity of older adults [10,13]. It has also been demonstrated to be useful for evaluating the functional capacity of patients with cardiovascular diseases [14]. In this context, there is only one study demonstrating that the 1′-sit-to-stand test may be a good predictor of undernutrition in individuals ≥ 50 years old with peripheral artery disease [15]. However, we could not find any other study investigating the usefulness of the 1′-sit-to-stand test in healthy older adults in comparison with older adults suffering from undernutrition. Thus, the present study is demonstrating for the first time that healthy older adults under a hypocaloric diet, characterizing a state of undernutrition, display an impaired function capacity, demonstrated by a reduced number of sit-to-stand repetitions in the 1′ test. Most importantly, this reduction in functional capacity was followed by impaired oxygen desaturation. Oxygen desaturation is particularly important for the elderly, as it may contribute to various health complications, including sleep-disordered breathing [16], which may additionally result in cognitive decline [17], increased daytime drowsiness [18], and elevated mortality risk [18]. Beyond that, hypoxia is related to cellular DNA damage [19], inflammation [19], and other pathological processes [19]. Therefore, we emphasize the importance of these findings, not only scientifically, but also for physicians to evaluate the nutritional status of older adults, even when they seem to present characteristic complaints relating to an apparent health condition.

In this way, in the present study, older adults on a hypocaloric diet showed a higher body weight, body mass index, and fat mass, along with lower lean mass, reduced hand grip strength, and decreased functional capacity, as measured by the 1 min sit-to-stand test, characterizing an older adult developing sarcopenic obesity. Notably, the literature emphasizes that sarcopenia refers to the progressive loss of skeletal muscle mass and strength associated with aging. This process may be exacerbated by a hypocaloric diet, as the reduction in metabolically active muscle tissue can lower the resting metabolic rate. A lower metabolic rate means that even a hypocaloric diet may not induce sufficient energy expenditure to achieve weight or fat loss. In fact, a large study performed with 3937 individuals aged 40 or older reported the clinical characteristics observed in our study [20]. This means that our findings related to body composition and muscle strength and functional capacity in older adults under a hypocaloric diet agree with the literature [20]. A hypocaloric diet can also trigger fat-preserving mechanisms due to metabolic adaptations. The body reduces energy expenditure and favors storing fat for survival, particularly in older adults, who have a slower metabolic response [20].

The cardiovascular hemodynamic response is typically impaired through the years in older adults, including structural and functional alterations [5]. Such impairments in cardiovascular health [21], also presented in the pulmonary [22] response, can be prevented by a healthy lifestyle, which includes the regular practice of physical activity. In fact, some studies have identified that undernutrition is highly prevalent among older adults presenting with heart diseases [1,2,3,4,15]. In the present study, several parameters obtained from the impedance cardiography revealed that older adults under a hypocaloric regimen presented a better stroke volume. Such a result can be at least partially attributed to an observed lower body weight and body fat, a phenomenon that was already described in another study [23]. Reduced body weight and body fat results in improved vascular resistance and, consequently, in a reduced cardiac workload, thus improving the stroke volume, as described previously [23]. In fact, beyond the lower body weight and better stroke volume, the hypocaloric group also presented with a reduced systemic vascular resistance (SVR) [24]. Improving the SVR is essential, particularly in elderly individuals, as elevated SVR contributes to hypertension and cardiovascular diseases, which are very prevalent among the older population [25]. Aging is associated with increased arterial stiffness and reduced vascular compliance, both of which elevate the SVR, leading to a heightened cardiac workload and potential left ventricular hypertrophy due to SVR elevation, which is underlined by impaired tissue perfusion, which can exacerbate age-related conditions such as peripheral artery disease and cognitive decline [25]. Beyond that, elevated arterial elastance is linked to aging. As individuals age, their central arteries experience dilation, resulting in thicker and stiffer walls [26,27]. Therapeutic strategies to improve SVR, including lifestyle changes, have been shown to reduce cardiovascular morbidity and mortality in the elderly [28]. Improving vascular health can thus enhance the quality of life and reduce the burden of cardiovascular events.

The cardiovascular hemodynamic response may be regulated/dysregulated by changes in the immune response [29]. Increased levels of IL-6 have been associated with negative outcomes in individuals, both with and without pre-existing cardiovascular disease (CVD) [29]. In fact, elevated levels of plasma IL-6 are consistently linked to poorer cardiovascular outcomes and higher all-cause mortality across various racial and ethnic groups [29]. In the present study, we did not find any differences in the cardiovascular hemodynamics for the IL-6 levels when comparing the normocaloric and hypocaloric groups. Such a finding has been previously demonstrated, although no plausible explanation was found by either the present study or by other studies [30]. This means that further studies are needed to clarify whether an acute and chronic hypocaloric dietetic regimen in older adults may affect the levels of IL-6. In addition, the analysis of TNF-alpha, another pro-inflammatory cytokine, revealed no differences in the cardiovascular hemodynamic parameters between the normocaloric and hypocaloric groups. Indeed, TNF-α plays a multifaceted role in cardiovascular hemodynamics, influencing the vascular tone, myocardial contractility, and cardiac remodeling [31]. While it can mediate protective immune responses, its overexpression in pathological conditions leads to deleterious effects, contributing to the progression of heart failure, atherosclerosis, and other cardiovascular diseases [31]. The therapeutic modulation of TNF-α remains a promising but challenging approach, highlighting the need for a deeper understanding of its precise roles in different stages of cardiovascular disease [31]. Further research into selective TNF-α inhibitors and their impact on cardiovascular hemodynamics may hold the key to novel treatments for cardiovascular disorders [31]. A hypocaloric diet has been associated with increased lifespan and reduced inflammation in animal models [32]. In humans, a hypocaloric diet has been shown to lower TNF-α levels and reduce markers of oxidative stress, potentially slowing the aging process [31]. However, the application of a hypocaloric diet in older adults requires careful consideration, as excessive restriction may lead to nutrient deficiencies, muscle loss, and a decline in quality of life [33]. Therefore, additional studies are required to better understand the effects and the underlying mechanisms involved in hypocaloric-diet-induced TNF-alpha-driven cardiovascular hemodynamic regulation in older adults.

After the discussion of pro-inflammatory cytokines, notably IL-6 and TNF-alpha, this paragraph aims to discuss the role of the anti-inflammatory cytokine IL-10 and the anti-inflammatory and anti-aging protein Klotho. IL-10 is a potent anti-inflammatory cytokine that plays a significant role in cardiovascular health, particularly in older adults, who often experience chronic inflammation and associated cardiovascular disorders [34]. Elevated levels of IL-10 have been shown to improve endothelial function, reduce vascular stiffness, and mitigate the adverse effects of inflammation on cardiovascular hemodynamics [35]. In aging populations, where pro-inflammatory cytokines like TNF-α and IL-6 are often elevated, IL-10 helps maintain a balance by inhibiting excessive inflammatory responses and promoting vascular health [12]. This protective mechanism may lower the risk of atherosclerosis and heart failure, contributing to better cardiovascular outcomes in older adults [12]. Furthermore, strategies to boost IL-10 levels, such as lifestyle interventions or specific dietary changes, may have therapeutic potential for enhancing cardiovascular health in this vulnerable population [12,22,36,37]. In addition, the protein klotho, which possesses anti-inflammatory, anti-aging, anti-fibrotic, antioxidant, and anti-cancer properties, was also investigated, although no differences between the normocaloric and hypocaloric groups were found. The relationship between Klotho and cardiovascular hemodynamics in older adults is complex. Reduced Klotho levels have been linked to several hemodynamic alterations, including increased arterial stiffness and elevated blood pressure [38]. Arterial stiffness is a significant predictor of cardiovascular events in older populations, and studies have demonstrated that a Klotho deficiency correlates with increased pulse wave velocity, a measure of arterial stiffness [39]. This relationship highlights Klotho’s potential role as a biomarker for cardiovascular health and a therapeutic target for improving hemodynamic function. However, due to the results observed in the present study, further studies investigating the influence of a hypocaloric diet on older adults are urgently needed.

Although the nutritional classification of individuals using the 24 h food record questionnaire and the food frequency questionnaire is accepted as the gold standard in nutritional evaluation, this method may present a bias, as the interviewee may not fully understand all the questions asked by the interviewer.

## 5. Conclusions

In conclusion, long-term hypocaloric diets in eutrophic older adults lead to an enhanced cardiovascular hemodynamic response but are associated with reduced functional capacity. This includes a diminished response in oxygen saturation during physical exertion, while not significantly affecting the cellular and humoral systemic immune responses.

## Figures and Tables

**Table 1:** Volunteer characteristics.

	Normocaloric	Hypocaloric	*p* Value
**Age (years)**	67.55 ± 6.02	68.17 ± 6.07	0.7239
**Body weight (kg)**	63.38 ± 11.58	75.63 ± 15.25 *	0.0039
**Height (m)**	1.59 ± 0.066	1.59 ± 0.87	0.9348
**BMI (kg/m^2^)**	25.56 ± 4.51	29.62 ± 4.56 *	0.0027
**SBP (mmHg)**	133.77 ± 24.17	133.32 ± 20.19	0.9414
**DBP (mmHg)**	78.83 ± 16.22	77.90 ± 11.57	0.8055
**HR (bpm)**	66.61 ± 10.90	71.04 ± 11.10	0.1648
**Fat mass (%)**	30.93 ± 12.56	39.86 ± 8.47 *	0.0025
**Lean mass (%)**	28.48 ± 6.58	24.86 ± 4.08 *	0.0073
**Waist (cm)**	85.33 ± 9.05	91.8 ± 9.19 *	0.0027
**Hip (cm)**	93.01 ± 16.28	105.28 ± 10.18 *	0.0009
**Waist-to-hip ratio**	0.92 ± 0.27	0.86 ± 0.15	0.5057
**Right calf (cm)**	34.89 ± 3.63	35.96 ± 3.32	0.5282
**Left calf (cm)**	35.25 ± 4.45	35.98 ± 3.10	0.9526
**Hand grip strength (kg)**	**26.93 ± 2.61**	23.59 ± 5.41 *	**0.0089**

kg = kilogram; m = meter; BMI = body mass index; SBP = systolic blood pressure; DBP = diastolic blood pressure; HR = heart rate; kg/m^2^ = kilogram per square meter; mmHg = millimeter of mercury; bpm = beats per minute; % = percentage; cm = centimeter. * Means statistically significant.

## Data Availability

The data that support the findings of this study are available from the corresponding author upon reasonable request.

## References

[1] Viart P. (1977). Hemodynamic findings in servere protein-calorie malnutrition. Am. J. Clin. Nutr..

[2] Wleklik M., Uchmanowicz I., Jankowska-Polańska B., Andreae C., Regulska-Ilow B. (2018). The Role of Nutritional Status in Elderly Patients with Heart Failure. J. Nutr. Health Aging..

[3] Narumi T., Arimoto T., Funayama A., Kadowaki S., Otaki Y., Nishiyama S., Takahashi H., Shishido T., Miyashita T., Miyamoto T. (2013). Prognostic importance of objective nutritional indexes in patients with chronic heart failure. J. Cardiol..

[4] Gámez-López A.L., Bonilla-Palomas J.L., Anguita-Sánchez M., Moreno-Conde M., López-Ibáñez C., Alhambra-Expósito R., Castillo-Domínguez J.C., Villar-Ráez A., Suárez de Lezo J. (2014). Rationale and design of PICNIC study: Nutritional intervention program in hospitalized patients with heart failure who are malnourished. Rev. Esp. Cardiol..

[5] Strait J.B., Lakatta E.G. (2012). Aging-associated cardiovascular changes and their relationship to heart failure. Heart Fail. Clin..

[6] Lisowska K.A., Storoniak H., Soroczyńska-Cybula M., Maziewski M., Dębska-Ślizień A. (2022). Serum Levels of α-Klotho, Inflammation-Related Cytokines, and Mortality in Hemodialysis Patients. J. Clin. Med..

[7] Kirkpatrick S.I., Subar A.F., Douglass D., Zimmerman T.P., Thompson F.E., Kahle L.L., George S.M., Dodd K.W., Potischman N. (2014). Performance of the Automated Self-Administered 24-hour Recall Relative to a Measure of True Intakes and to an Interviewer-Administered 24-h Recall. Am. J. Clin. Nutr..

[8] Yaghi N., Boulos C., Baddoura R., Abifadel M., Yaghi C. (2022). Validity and reliability of a food frequency questionnaire for community dwelling older adults in a Mediterranean country: Lebanon. Nutr. J..

[9] Salles-Dias L.P., Brandao-Rangel M.A.R., Cristina-Rosa A., Morais-Felix R.T., Oliveira-Freitas S., Oliveira L.V.F., Moraes-Ferreira R., Bachi A.L.L., Coutinho E.T., Frison C.R. (2024). Functional analysis of airway remodeling is related with fibrotic mediators in asthmatic children. J. Asthma..

[10] Strassmann A., Steurer-Stey C., Lana K.D., Zoller M., Turk A.J., Suter P., Puhan M.A. (2013). Population-based reference values for the 1-min sit-to-stand test. Int. J. Public Health.

[11] Cheung C.H., Khaw M.L., Tam V.C.W., Ying M.T.C., Lee S.W.Y. (2020). Performance evaluation of a portable bioimpedance cardiac output monitor for measuring hemodynamic changes in athletes during a head-up tilt test. J. Appl. Physiol..

[12] Silva-Reis A., Rodrigues Brandao-Rangel M.A., Moraes-Ferreira R., Gonçalves-Alves T.G., Souza-Palmeira V.H., Aquino-Santos H.C., Bachi A.L.L., de Oliveira L.V.F., Lopes-Martins R.Á.B., Oliveira-Silva I. (2022). Combined resistance and aerobic training improves lung function and mechanics and fibrotic biomarkers in overweight and obese women. Front. Physiol..

[13] Bohannon R.W., Crouch R. (2019). 1-Minute Sit-to-Stand Test: Systematic review of procedures, performance, and clinimetric properties. J. Cardiopulm. Rehabil. Prev..

[14] Fuentes-Abolafio I.J., Bernal-López M.R., Gómez-Huelgas R., Ricci M., Cuesta-Vargas A.I., Pérez-Belmonte L.M. (2022). Relationship between quadriceps femoris muscle architecture and muscle strength and physical function in older adults with heart failure with preserved ejection fraction. Sci. Rep..

[15] Carvalho J., Correia M.A., Kanegusuku H., Longano P., Wolosker N., Ritti-Dias R.M., Cucato G.G. (2022). Association between the risk of malnutrition and functional capacity in patients with peripheral arterial disease: A cross-sectional study. PLoS ONE.

[16] Kitakata H., Kohno T., Fukuda K. (2018). Sleep-disordered breathing in the elderly: Is it distinct from that in the younger or middle-aged populations?. J. Thorac. Dis..

[17] Snyder B., Simone S.M., Giovannetti T., Floyd T.F. (2021). Cerebral Hypoxia: Its Role in Age-Related Chronic and Acute Cognitive Dysfunction. Anesth. Analg..

[18] Gooneratne N.S., Richards K.C., Joffe M., Lam R.W., Pack F., Staley B., Dinges D.F., Pack A.I. (2011). Sleep disordered breathing with excessive daytime sleepiness is a risk factor for mortality in older adults. Sleep.

[19] Wei Y., Giunta S., Xia S. (2022). Hypoxia in Aging and Aging-Related Diseases: Mechanism and Therapeutic Strategies. Int. J. Mol. Sci..

[20] Yoo S., Kim D.Y., Lim H. (2020). Sarcopenia in relation to nutrition and lifestyle factors among middle-aged and older Korean adults with obesity. Eur. J. Nutr..

[21] Wolsk E., Bakkestrøm R., Thomsen J.H., Balling L., Andersen M.J., Dahl J.S., Hassager C., Møller J.E., Gustafsson F. (2017). The Influence of Age on Hemodynamic Parameters During Rest and Exercise in Healthy Individuals. JACC Heart Fail..

[22] Brandao-Rangel M.A.R., Brill B., de Souza Carvalho E., Melamed D., Moraes-Ferreira R., Silva-Reis A., Leonardo P.S., Frison C.R., De Angelis K., Vieira R.P. (2024). Physically Active Lifestyle Attenuates Impairments on Lung Function and Mechanics in Hypertensive Older Adults. Adv. Respir. Med..

[23] Lavie C.J., Milani R.V., Ventura H.O. (2009). Obesity and cardiovascular disease: Risk factor, paradox, and impact of weight loss. J. Am. Coll. Cardiol..

[24] Theodoridis X., Chourdakis M., Papaemmanouil A., Chaloulakou S., Georgakou A.V., Chatzis G., Triantafyllou A. (2024). The Effect of Diet on Vascular Aging: A Narrative Review of the Available Literature. Life.

[25] Iadecola C., Yaffe K., Biller J., Bratzke L.C., Faraci F.M., Gorelick P.B., Gulati M., Kamel H., Knopman D.S., Launer L.J. (2016). Impact of Hypertension on Cognitive Function: A Scientific Statement From the American Heart Association. Hypertension.

[26] Najjar S.S., Scuteri A., Lakatta E.G. (2005). Arterial aging: Is it an immutable cardiovascular risk factor?. Hypertension.

[27] Sonaglioni A., Baravelli M., Lombardo M., Sommese C., Anzà C., Kirk J.A., Padeletti L. (2018). Ventricular-arterial coupling in centenarians without cardiovascular diseases. Aging Clin. Exp. Res..

[28] Kähönen E., Aatola H., Lehtimäki T., Haarala A., Sipilä K., Juonala M., Raitakari O.T., Kähönen M., Hutri-Kähönen N. (2021). Influence of early life risk factors and lifestyle on systemic vascular resistance in later adulthood: The cardiovascular risk in young Finns study. Blood Press..

[29] Khan M.S., Talha K.M., Maqsood M.H., Rymer J.A., Borlaug B.A., Docherty K.F., Pandey A., Kahles F., Cikes M., Lam C.S.P. (2024). Interleukin-6 and Cardiovascular Events in Healthy Adults: MESA. JACC Adv..

[30] Arabi Y., Jawdat D., Bouchama A., Tamim H., Tamimi W., Al-Balwi M., Al-Dorzi H.M., Sadat M., Afesh L., Abdullah M.L. (2019). Permissive underfeeding, cytokine profiles and outcomes in critically ill patients. PLoS ONE.

[31] Padfield G.J., Din J.N., Koushiappi E., Mills N.L., Robinson S.D., Cruden Nle M., Lucking A.J., Chia S., Harding S.A., Newby D.E. (2013). Cardiovascular effects of tumour necrosis factor α antagonism in patients with acute myocardial infarction: A first in human study. Heart.

[32] Zhang C., Wu J., Xu X., Potter B.J., Gao X. (2010). Direct relationship between levels of TNF-alpha expression and endothelial dysfunction in reperfusion injury. Basic. Res. Cardiol..

[33] Serra M.C., Beavers D.P., Henderson R.M., Kelleher J.L., Kiel J.R., Beavers K.M. (2019). Effects of a Hypocaloric, Nutritionally Complete, Higher Protein Meal Plan on Regional Body Fat and Cardiometabolic Biomarkers in Older Adults with Obesity. Ann. Nutr. Metab..

[34] Krishnamurthy P., Rajasingh J., Lambers E., Qin G., Losordo D.W., Kishore R. (2009). IL-10 inhibits inflammation and attenuates left ventricular remodeling after myocardial infarction via activation of STAT3 and suppression of HuR. Circ. Res..

[35] Stafford N., Assrafally F., Prehar S., Zi M., De Morais A.M., Maqsood A., Cartwright E.J., Mueller W., Oceandy D. (2020). Signaling via the Interleukin-10 Receptor Attenuates Cardiac Hypertrophy in Mice During Pressure Overload, but not Isoproterenol Infusion. Front. Pharmacol..

[36] Kondo H., Abe I., Gotoh K., Fukui A., Takanari H., Ishii Y., Ikebe Y., Kira S., Oniki T., Saito S. (2018). Interleukin 10 Treatment Ameliorates High-Fat Diet-Induced Inflammatory Atrial Remodeling and Fibrillation. Circ. Arrhythm. Electrophysiol..

[37] Pires D.A., Brandão-Rangel M.A.R., Silva-Reis A., Olímpio F.R.S., Aimbire F., Oliveira C.R., Mateus-Silva J.R., Zamarioli L.S., Bachi A.L.L., Bella Y.F. (2024). Vitamin C Inhibits Lipopolysaccharide-Induced Hyperinflammatory State of Chronic Myeloid Leukemia Cells through Purinergic Signaling and Autophagy. Nutrients.

[38] Abraham C.R., Li A. (2022). Aging-suppressor Klotho: Prospects in diagnostics and therapeutics. Ageing Res. Rev..

[39] Akhiyat N., Ozcan I., Gulati R., Prasad A., Tchkonia T., Kirkland J.L., Lewis B., Lerman L.O., Lerman A. (2024). Patients With Coronary Microvascular Dysfunction Have Less Circulating α-Klotho. J. Am. Heart Assoc..

